# Correction: A supramolecular strategy for tuning the energy level of naphthalenediimide: promoted formation of radical anions with extraordinary stability

**DOI:** 10.1039/c5sc90039e

**Published:** 2015-07-01

**Authors:** Qiao Song, Fei Li, Zhiqiang Wang, Xi Zhang

**Affiliations:** a The Key Lab of Organic Optoelectronics & Molecular Engineering , Department of Chemistry , Tsinghua University , Beijing 100084 , P. R. China . Email: xi@mail.tsinghua.edu.cn

## Abstract

Correction for ‘A supramolecular strategy for tuning the energy level of naphthalenediimide: promoted formation of radical anions with extraordinary stability’ by Qiao Song *et al.*, *Chem. Sci.*, 2015, **6**, 3342–3346.



## 

The authors regret the incorrect calculation of LUMO and HOMO values in the manuscript. It should be LUMO = (–4.40 – *E*_1_) eV against vacuum.

The LUMO and HOMO values in Fig. 4 should be as following.

**Fig. 4:**
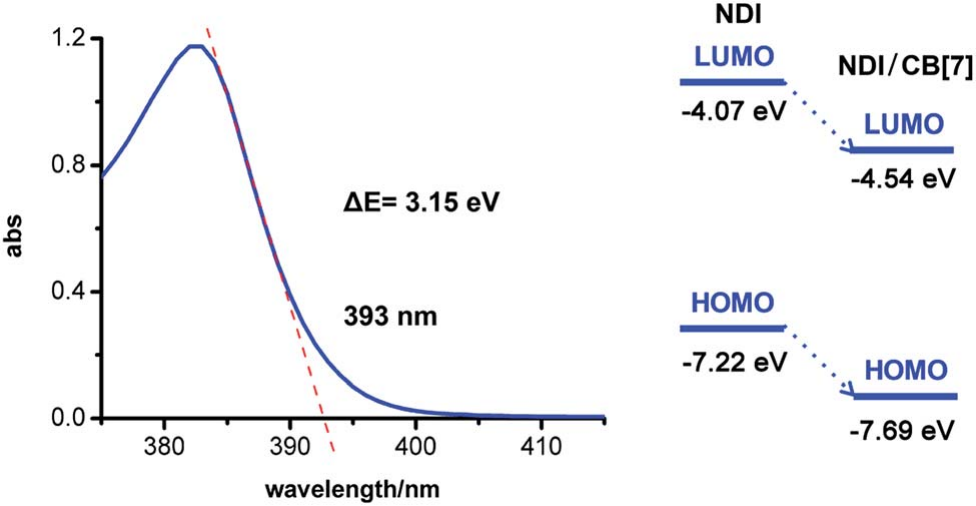
Calculation of the energy gap between the HOMO and LUMO of NDI and NDI/CB[7] (left); LUMO and HOMO energy of NDI and NDI/CB[7] (right).

The corresponding text should also be corrected as “From the calculated LUMO and HOMO energy shown in Fig. 4, the LUMO energy of NDI/CB[7] is 0.47 eV lower than NDI itself, and therefore the LUMO energy level is as low as –4.54 eV.”

Fig. S10 in ESI should be corrected as:

**Fig. S10:**
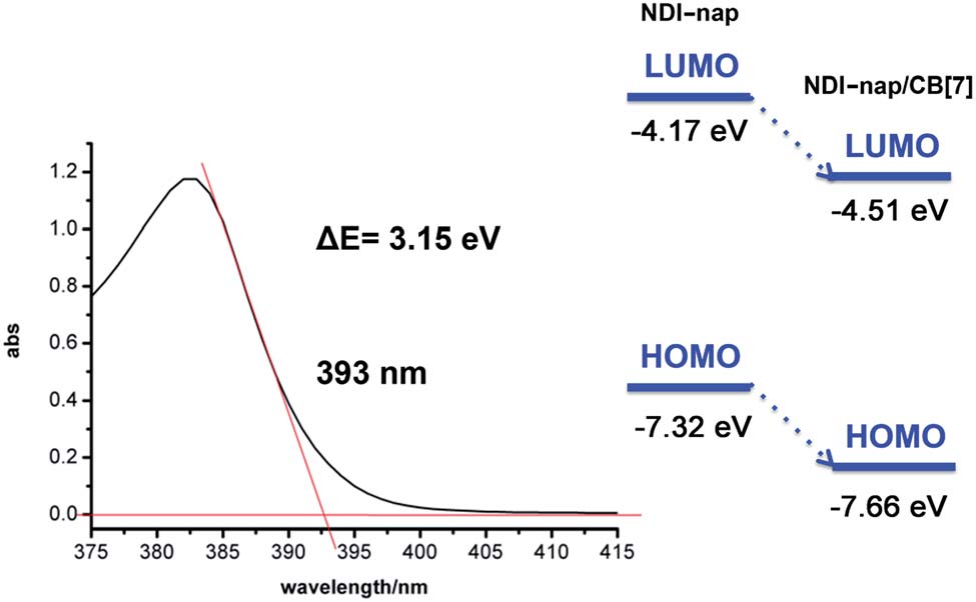
Calculation of band gap between HOMO and LUMO of NDI-nap and NDI-nap/CB[7] (left); LUMO and HOMO energy of NDI-nap and NDI-nap/CB[7] (right).

The Royal Society of Chemistry apologises for these errors and any consequent inconvenience to authors and readers.

